# Revisiting the Watching-Eyes Effect in the Ultimatum Game: The Role of Public Self-Awareness

**DOI:** 10.3390/bs16071177

**Published:** 2026-07-13

**Authors:** Jieyu Lv, Jinghan Zhang, Chujian Zhang, Yingjun Zhang

**Affiliations:** 1 Department of Psychology, School of Sociology and Psychology, Central University of Finance and Economics, Beijing 100081, China; 18189520996@163.com (J.Z.); 13659431381@163.com (C.Z.); 2 Mental Health Education and Counseling Center, Beijing Normal University, Beijing 100875, China; yjzhang@bnu.edu.cn

**Keywords:** watching-eyes effect, fair decision-making, ultimatum game, dispositional public self-awareness, state public self-awareness

## Abstract

Fair decision-making is a critical topic in social psychology, yet the conditions under which visual social-presence cues influence fairness remain unclear. Using two online Ultimatum Game studies, the present research examined whether eye cues affect proposers’ allocation behavior and responders’ rejection behavior, and whether dispositional or state public self-awareness serves as a boundary condition. Study 1 adopted a three-factor design covering cue type, dispositional public self-awareness and repeated decision rounds. No consistent cue-related tendency appeared for proposers’ average resource offers, exact equal split choices or responders’ unequal offer rejection. Trait public self-awareness, decision round, and the interaction between each pair of variables showed no meaningful influence on all measured fairness indicators. Study 2 adopted a two-factor design with cue type and experimentally induced state public self-awareness. Among all fairness indicators, only a weak tendency for equal allocation choices differed between cue groups, and this trend went against mainstream predictions of prosocial observation effects. No consistent cue-related tendency existed for average allocation or responders’ rejection choices, and state public self-awareness did not interact with cue type to shape participants’ decisions. Taken together, the whole set of observations shows little consistent support for the watching-eyes effect in online Ultimatum Game contexts.

## 1. Introduction

Fair decision-making is a core issue in social psychology and behavioral decision-making because many everyday social interactions require individuals to balance personal benefit against equality, reciprocity, and others’ evaluations. The Ultimatum Game provides a useful paradigm for examining such trade-offs because it separates two interdependent roles: proposers decide how to divide a resource, whereas responders decide whether to accept or reject the proposed allocation (Güth et al., 1982; Sanfey et al., 2003). This paradigm has therefore been widely used to investigate fairness norms, reciprocal punishment, and social preferences in economic and interpersonal decision-making (Camerer, 2003; Thaler, 1988). Importantly, fairness in the Ultimatum Game is not a single unitary outcome. It can be reflected in the continuous amount offered by proposers, in whether proposers choose an exact equal split, and in whether responders reject unequal offers. These indicators are theoretically related but behaviorally distinct, because proposer decisions mainly reflect allocation preferences, whereas responder decisions reflect reactions to perceived unfairness. Accordingly, examining multiple fairness-related outcomes can provide a more precise understanding of whether a social cue influences fair decision-making broadly or only affects a specific decision role or behavioral indicator.

One social cue that has received sustained attention is the watching-eyes effect. Eye-like stimuli are often assumed to imply potential observation and, as a result, may activate reputation concerns, social-norm compliance, or prosocial motivation (Baillon et al., 2013; Bateson et al., 2006; Haley & Fessler, 2005; Rigdon et al., 2009). From this perspective, eye cues may make people feel that their behavior is being monitored, thereby encouraging decisions that appear fairer or more socially acceptable. However, the reliability and boundary conditions of the watching-eyes effect remain debated. Some studies have found that eye cues increase generosity or cooperation, whereas other studies and replication attempts have reported weak, inconsistent, or null effects (Fehr & Schneider, 2010; Oda, 2019; Sparks & Barclay, 2013, 2015). Meta-analytic and replication evidence further suggests that artificial surveillance cues do not uniformly promote prosocial behavior and that their effects may depend on the task, sample, setting, and outcome definition (Dear et al., 2019; Nettle et al., 2013; Northover et al., 2017a, 2017b; Rotella et al., 2021; Tane & Takezawa, 2011). This debate is especially relevant for online experimental contexts, where participants are physically anonymous and eye cues may not create the same level of real social observation as in face-to-face settings. Therefore, rather than assuming a universally prosocial effect of eye cues, it is necessary to test whether such cues influence specific fairness-related behaviors in online Ultimatum Game tasks. Against this backdrop, we proposed Hypothesis 1: Eye cues will increase fairness-related decisions in online Ultimatum Game tasks.

Public self-awareness may be a theoretically relevant boundary condition for the watching-eyes effect. Public self-awareness refers to the tendency or state of attending to oneself as a social object who can be observed and evaluated by others (Fenigstein et al., 1975; Froming et al., 1982; Prentice-Dunn & Rogers, 1982). Individuals with higher public self-awareness may be more sensitive to social-evaluative cues because they are more likely to consider how their behavior appears to others. Similarly, temporarily priming public self-awareness may increase attention to one’s public image and thereby strengthen responses to cues that imply observation (Brockner et al., 1982; Keller & Pfattheicher, 2011; Pfattheicher & Keller, 2015; Van Bommel et al., 2012). If eye cues function by making potential observation or social evaluation salient, then individuals with higher dispositional public self-awareness or experimentally heightened state public self-awareness may show stronger fairness-related responses to such cues (Gervais & Norenzayan, 2012). Nevertheless, the moderating role of public self-awareness has not been established consistently, particularly in anonymous online economic-game settings where real reputational consequences are limited. This makes it important to examine both dispositional and state forms of public self-awareness as possible boundary conditions of the watching-eyes effect. To address this issue, we proposed Hypothesis 2: Public self-awareness will moderate the influence of eye cues on fairness-related decisions, such that the effect of eye cues would be stronger among individuals with higher dispositional public self-awareness or under experimentally primed state public self-awareness.

The present research re-examined the relationship among eye cues, public self-awareness, and fairness-related decisions in two online Ultimatum Game studies. Study 1 tested cue type, dispositional public self-awareness, and decision round using a mixed design. Study 2 tested cue type and experimentally primed state public self-awareness using a between-subjects design. Across the two studies, we distinguished three focal outcomes: proposers’ mean allocation amount, proposers’ strict 5:5 allocation offers, and responders’ rejection of non-5:5 offers. This design allowed us to evaluate whether eye cues exert a general influence on fair decision-making or whether any apparent effect is limited to a particular outcome, role, or experimental context. More broadly, by directly examining corrected outcome-specific evidence in online settings, the present research contributes to ongoing discussions about the robustness and boundary conditions of the watching-eyes effect.

## 2. Study 1: The Effects of Dispositional Public Self-Awareness and Cue Type on Fair Decision-Making

### 2.1. Method

#### 2.1.1. Participants

Sample size was determined a priori using G*Power 3.1 software (Faul et al., 2007). With an effect size f=0.15 and significance level α=0.05, 128 participant pairs (256 total participants) were required to achieve 1−β=0.80 statistical power. During initial data checking, one missing value was found for age and gender, respectively. To ensure data integrity, the mean of non-missing values was used to replace the missing age value, and the mode of non-missing values replaced the missing gender value. Finally, 260 valid participants (mean age = 21.95 ± 2.61, 145 males, 115 females) were included. The study was conducted in accordance with the Declaration of Helsinki and approved by the Ethics Committee of Central University of Finance and Economics (Approval No. IRB20260506002, approved on 6 May 2026). This low-risk online experiment used fully anonymized data without sensitive information or potential harm to participants, so it qualifies for ethical review exemption per national regulations. Written informed consent was obtained from all participants, who joined voluntarily and could withdraw freely. All experimental data were anonymized.

#### 2.1.2. Design

Study 1 used a 2 (cue type: eye cues vs. cloud cues) × 2 (dispositional public self-awareness: high vs. low) × 2 (round: communication vs. no-communication) mixed design. Cue type and dispositional public self-awareness were between-subject factors, and round was the within-subject factor. The dependent variables were proposers’ mean allocation amount, proposers’ proportion of strict 5:5 allocation offers, and responders’ rejection rate of non-5:5 offers.

#### 2.1.3. Materials

##### Types of Cues

**Figure 1:**
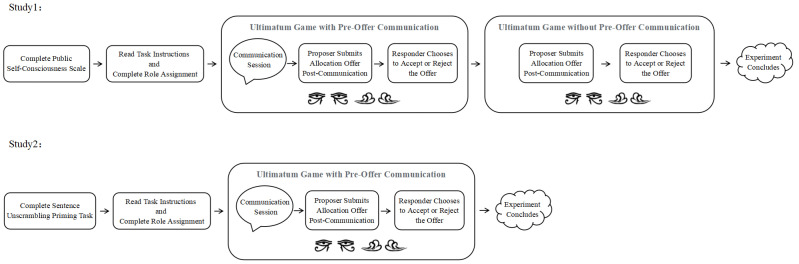
Research flowchart.

The eye cue was an image of the Eye of Horus (Haley & Fessler, 2005), and the control cue was a cloud image (Sun et al., 2020). The two images were used as the visual cue manipulation and were displayed only on the computer task pages, as illustrated in the experimental flowchart (Figure 1). In the eye-cue condition, the eye image appeared on the relevant oTree (Chen et al., 2016) task page; in the cloud-cue condition, the cloud image appeared in the same task-page position. To reduce demand characteristics, participants received a cover instruction about the meaning of the pictures rather than being told that the images were intended to induce surveillance or observation.

##### Dispositional Public Self-Awareness Scale

The Public Self-Awareness Scale (Fenigstein et al., 1975) was used, including 10 items such as “I care about what others think of me”, “I am always worried about making a good impression”, etc. Items were rated on a 5-point scale (1 = *strongly disagree* to 5 = *strongly agree*). The 10 items were summed to form a total dispositional public self-awareness score. Participants were divided into high and low dispositional public self-awareness groups using the sample mean as the cut-off (*M* = 37.13): participants scoring above the sample mean were assigned to the high group (*n* = 164), whereas participants scoring below the sample mean were assigned to the low group (*n* = 96). This mean-split procedure accounts for the unequal group sizes and was used consistently in all Study 1 analyses.

##### Ultimatum Game

The Ultimatum Game involved two roles: proposer and responder. The proposer decided how to split 10 RMB with the responder, and the responder could accept or reject the offer. Following prior Ultimatum Game research that treats an equal 5:5 split as a fair offer, the present manuscript uses the term “strict 5:5 allocation offers” to refer specifically to exact equal splits. Non-5:5 offers were used to calculate responders’ rejection rate of unequal offers, but this binary indicator was interpreted alongside the continuous allocation-amount analysis to avoid treating near-equal and highly unequal offers as psychologically identical (Sanfey et al., 2003).

#### 2.1.4. Procedure

The overall research process is shown in Figure 1. Study 1 was conducted entirely online through the oTree platform (Chen et al., 2016). Participants were recruited through online posters and were invited into a shared WeChat group, where they accessed the experiment by clicking the oTree link on their own computers. After providing informed consent, participants completed the Dispositional Public Self-Awareness Scale and were then randomly assigned to the eye-cue or cloud-cue condition. Participants were randomly paired within each cue condition, and one participant in each dyad was randomly assigned as the proposer, while the other was assigned as the responder. Roles remained fixed across the two rounds.

The cue manipulation was embedded in the computer task pages. In the first round, which included communication, the assigned eye or cloud image was first displayed on the communication page together with a three-minute countdown. After the countdown ended, proposers entered the allocation page, where the same cue image was displayed for a second time while they decided how to divide the 10 RMB. Responders first waited for the proposer to complete the allocation and then entered the acceptance/rejection page, where the same cue image was again displayed while they decided whether to accept or reject the offer. In the second round, which did not include communication, the cue image was displayed directly on the proposer allocation page and on the responder acceptance/rejection page. Thus, the eye and cloud cues were presented visually on the computer operation pages during the relevant communication and decision stages, but they were not presented as real-time observation by another person.

#### 2.1.5. AI Tools Declaration

During the preparation of this manuscript, the authors used ChatGPT (GPT-5.5 Thinking model) by OpenAI and other large language model tools solely for language polishing, grammar checking, and improving readability. These tools were not employed for study design, data collection, statistical analysis, or interpretation of the results. All AI-generated suggestions were reviewed and edited by the authors, who take full responsibility for the final content of the manuscript.

### 2.2. Results

We adopted a three-factor mixed-design ANOVA for Study 1. Cue type and dispositional public self-awareness were treated as between-subject factors, and round was treated as the within-subject factor. Descriptive statistics are presented in Table 1.

#### 2.2.1. Manipulation Check for Dispositional Public Self-Awareness Grouping

Dispositional public self-awareness was computed by summing the ten public self-awareness items. Participants were divided into high and low dispositional public self-awareness groups using the sample-mean cut-off. This grouping variable was used as the between-subject public self-awareness factor in Study 1.

#### 2.2.2. Effects on Proposers’ Mean Allocation Amount

Collapsed across dispositional public self-awareness and round, proposers’ mean allocation amount was similar in the cloud-cue condition (M=5.52, SD=1.23) and the eye-cue condition (M=5.56, SD=1.46). The main effect of cue type was not significant, F(1, 126)=0.07, p=0.786, $\eta_{p}^{2}=0.001$. The main effect of dispositional public self-awareness was not significant, F(1, 126)=3.27, p=0.073, $\eta_{p}^{2}=0.025$. The main effect of round was not significant, F(1, 126)=1.39, p=0.241, $\eta_{p}^{2}=0.011$. The cue type × dispositional public self-awareness interaction was not significant, F(1, 126)=0.02, p=0.898, $\eta_{p}^{2}<0.001$; the cue type × round interaction was not significant, F(1, 126)=0.57, p=0.454, $\eta_{p}^{2}=0.004$; the dispositional public self-awareness × round interaction was not significant, F(1, 126)=0.70, p=0.403, $\eta_{p}^{2}=0.006$; and the three-way interaction was not significant, F(1, 126)=0.39, p=0.534, $\eta_{p}^{2}=0.003$.

**Figure 2:**
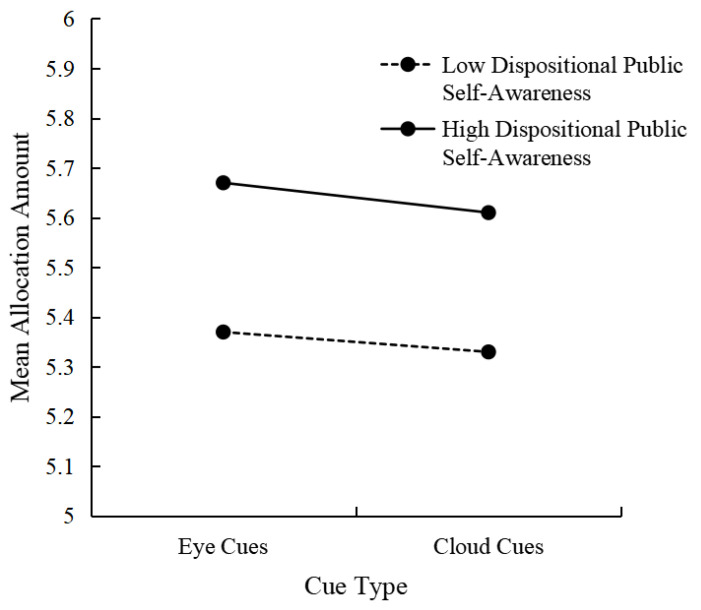
Study1 interaction plot between cue type and proposers’ dispositional public self-awareness (mean allocation amount).

For visualization, the Study 1 allocation interaction pattern is shown in Figure 2.

#### 2.2.3. Effects on Proposers’ Strict 5:5 Allocation Offers

For proposers’ strict 5:5 allocation offers, the descriptive proportion was M=0.73, SD=0.45 in the cloud-cue condition and M=0.68, SD=0.47 in the eye-cue condition. The main effect of cue type was not significant, F(1, 126)=0.49, p=0.486, $\eta_{p}^{2}=0.004$. The main effect of dispositional public self-awareness was not significant, F(1, 126)=1.53, p=0.218, $\eta_{p}^{2}=0.012$. The main effect of round was not significant, F(1, 126)=1.77, p=0.185, $\eta_{p}^{2}=0.014$. The cue type × dispositional public self-awareness interaction was not significant, F(1, 126)=0.01, p=0.912, $\eta_{p}^{2}<0.001$; the cue type × round interaction was not significant, F(1, 126)=0.23, p=0.629, $\eta_{p}^{2}=0.002$; the dispositional public self-awareness × round interaction was not significant, F(1, 126)=0.20, p=0.652, $\eta_{p}^{2}=0.002$; and the three-way interaction was not significant, F(1, 126)=0.16, p=0.694, $\eta_{p}^{2}=0.001$.

**Figure 3:**
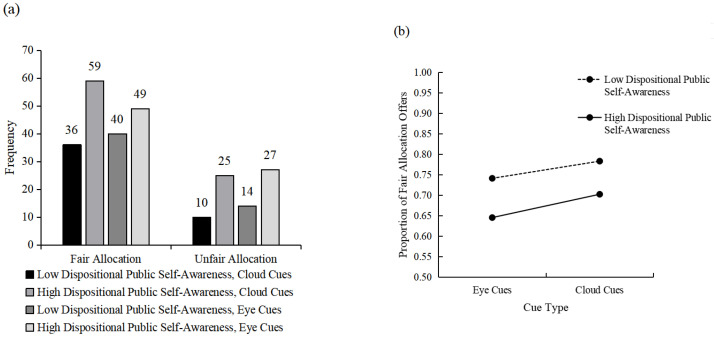
Study 1 interaction between cue type and proposers’ dispositional public self-awareness (strict 5:5 allocation offers): (**a**) Interaction plot, (**b**) interaction line graph.

For visualization, the Study 1 strict 5:5 allocation interaction pattern is shown in Figure 3.

#### 2.2.4. Effects on Responders’ Rejection of Non-5:5 Offers

Responders’ rejection of non-5:5 offers was coded from the recorded acceptance/rejection decision and the proposer offer in each round. The rejection proportion was M=0.08, SD=0.27 in the cloud-cue condition and M=0.08, SD=0.28 in the eye-cue condition. The main effect of cue type was not significant, F(1, 126)=0.00, p=0.979, $\eta_{p}^{2}<0.001$. The main effect of dispositional public self-awareness was not significant, F(1, 126)=0.03, p=0.855, $\eta_{p}^{2}<0.001$. The main effect of round was not significant, F(1, 126)=2.02, p=0.158, $\eta_{p}^{2}=0.016$. The cue type × dispositional public self-awareness interaction was not significant, F(1, 126)=0.33, p=0.567, $\eta_{p}^{2}=0.003$; the cue type × round interaction was not significant, F(1, 126)=0.00, p=0.974, $\eta_{p}^{2}<0.001$; the dispositional public self-awareness × round interaction was not significant, F(1, 126)=0.44, p=0.510, $\eta_{p}^{2}=0.003$; and the three-way interaction was not significant, F(1, 126)=0.53, p=0.468, $\eta_{p}^{2}=0.004$.

**Figure 4:**
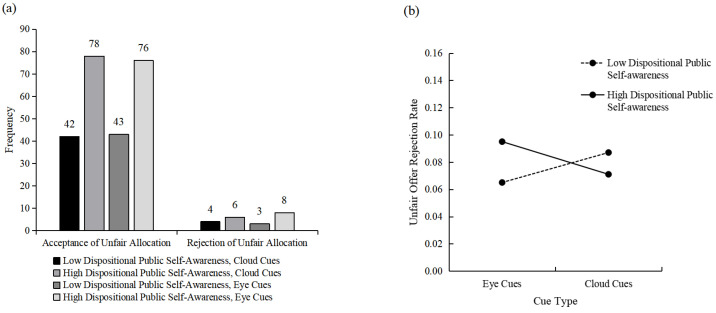
Study 1 interaction between cue type and responders’ dispositional public self-awareness (rejection of non-5:5 offers): (**a**) Interaction plot, (**b**) interaction line graph. The four experimental conditions are Cloud/Low PSA, Cloud/High PSA, Eye/Low PSA and Eye/High PSA, as shown in the figure legend.

For visualization, the Study 1 responder rejection interaction pattern is shown in Figure 4.

### 2.3. Discussion

The results did not support the proposed hypotheses. Cue type did not significantly affect proposers’ mean allocation amount, proposers’ strict 5:5 allocation offers, or responders’ rejection of non-5:5 offers. In addition, dispositional public self-awareness, round, and all interaction terms were not significant for the three focal outcomes. Thus, Study 1 provides no evidence that eye cues broadly influenced fair decision-making in the present online Ultimatum Game.

This null pattern is theoretically informative. It shows that eye cues exerted no consistent influence on the total amount participants allocated, their preference for equal splits, or their willingness to reject unequal offers. Dispositional public self-awareness also failed to act as a stable contextual factor that alters responses to visual cues. Therefore, Study 1 demonstrates that eye cues cannot produce widespread, predictable shifts in people’s fairness-related choices.

The absence of significant cue-type effects is consistent with prior work showing heterogeneous or null effects of artificial surveillance cues in economic games (Dear et al., 2019; Northover et al., 2017a, 2017b; Rotella et al., 2021; Tane & Takezawa, 2011). In online settings, eye cues may be too weak to produce credible perceived observation, especially when the task is anonymous and the social consequences of the decision are limited (Lamba & Mace, 2010).

## 3. Study 2: The Effects of State Public Self-Awareness and Cue Type on Fair Decision-Making

### 3.1. Method

#### 3.1.1. Participants

The final valid sample consisted of 256 participants ($M_{age}=21.93\pm 2.95$, 120 males, 136 females). The study followed the Declaration of Helsinki. This low-risk online experiment used fully anonymized data without sensitive information or potential harm to participants, so it qualifies for ethical review exemption per national regulations.

Informed consent was obtained from all participants before the experiment. All participants were informed of the purpose and procedures of the study, participated voluntarily, and could withdraw at any time without penalty. All data were collected anonymously.

#### 3.1.2. Design

Study 2 adopted a 2 (cue type: eye cues vs. cloud cues) × 2 (state public self-awareness: primed vs. unprimed) between-subjects design.

#### 3.1.3. Materials

State public self-awareness priming was adapted from Ding (2014). The manipulation was validated in a pilot study. The cue materials were the same as those used in Study 1. The eye-cue condition displayed the Eye of Horus image, whereas the cloud-cue condition displayed the cloud image. The images were displayed only on the computer task pages, as shown in the experimental flowchart (Figure 1). Participants received a cover instruction about the meaning of the pictures and were not explicitly told that the images were intended to represent surveillance or observation.

#### 3.1.4. Procedure

Study 2 was also conducted entirely online through the oTree platform (Chen et al., 2016). Participants were recruited through online posters and entered the experiment by clicking the oTree link. They first completed the state public self-awareness priming or control task and were then randomly assigned to the eye-cue or cloud-cue condition. Participants were randomly paired within condition, and one participant in each dyad was randomly assigned as the proposer, while the other was assigned as the responder.

The cue manipulation was implemented on the computer task pages in the same manner as in Study 1. In the communication stage, the assigned eye or cloud image appeared on the communication page. Proposers then viewed the assigned cue image again on the allocation page while deciding how to divide the 10 RMB. Responders viewed the same assigned cue image on the acceptance/rejection page while deciding whether to accept or reject the proposer’s offer. The pictures were therefore presented as visual elements of the online task interface rather than as actual observation by another person.

### 3.2. Results

Study 2 was analyzed using the experimentally manipulated state public self-awareness condition (primed vs. control), not a median split of the measured state-awareness score. Responders’ rejection of non-5:5 offers was recomputed from the recorded acceptance/rejection decision and the proposer offer. Descriptive statistics are presented in Table 2.

#### 3.2.1. Manipulation Check for State Public Self-Awareness

The priming manipulation increased state public self-awareness. Participants in the primed condition reported higher state public self-awareness (M=12.70, SD=2.25) than those in the control condition (M=11.66, SD=3.19), Welch’s t(228.15)=−3.03, p=0.003.

#### 3.2.2. Effects on Proposers’ Mean Allocation Amount

Collapsed across state public self-awareness condition, proposers’ mean allocation amount was similar in the cloud-cue condition (M=5.23, SD=0.74) and the eye-cue condition (M=5.19, SD=0.99). The main effect of cue type was not significant, F(1, 124)=0.07, p=0.789, $\eta_{p}^{2}=0.001$. The main effect of state public self-awareness priming was not significant, F(1, 124)=0.45, p=0.504, $\eta_{p}^{2}=0.004$. The cue type × state public self-awareness interaction was not significant, F(1, 124)=0.45, p=0.504, $\eta_{p}^{2}=0.004$.

**Figure 5:**
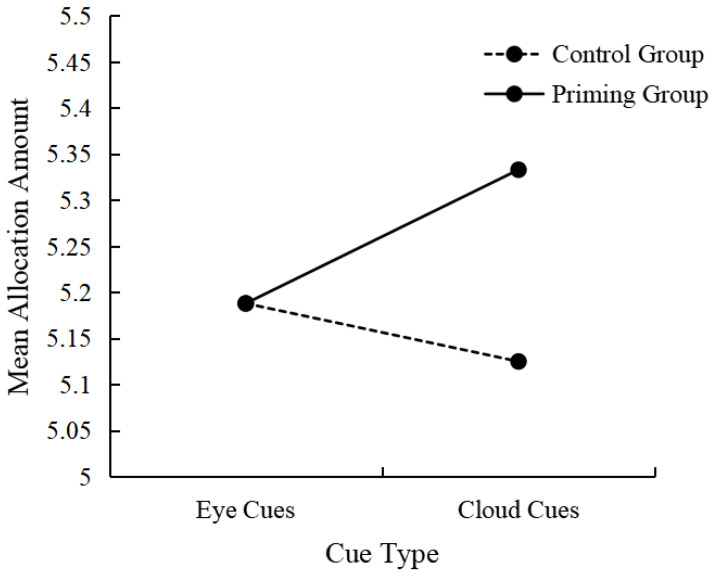
Study 2 interaction plot between cue type and proposers’ state public self-awareness (mean allocation amount).

For visualization, the Study 2 allocation interaction pattern is shown in Figure 5.

#### 3.2.3. Effects on Proposers’ Strict 5:5 Allocation Offers

For proposers’ strict 5:5 allocation offers, the cloud-cue condition showed a higher exact-equality proportion (M=0.89, SD=0.31) than the eye-cue condition (M=0.75, SD=0.44). The main effect of cue type was significant, F(1, 124)=4.32, p=0.040, $\eta_{p}^{2}=0.034$. Notably, this raw effect ran in the direction opposite to the standard prosocial-surveillance prediction: proposers under eye cues chose exact equal splits *less* frequently than proposers under cloud cues, not more. The main effect of state public self-awareness priming was not significant, F(1, 124)=0.48, p=0.490, $\eta_{p}^{2}=0.004$. The cue type × state public self-awareness interaction was not significant, F(1, 124)=0.05, p=0.818, $\eta_{p}^{2}<0.001$. Thus, a raw cue-type difference emerged for the binary indicator of exact equality; however, this isolated disparity fails to hold across all measured fairness outcomes in the two studies.

**Figure 6:**
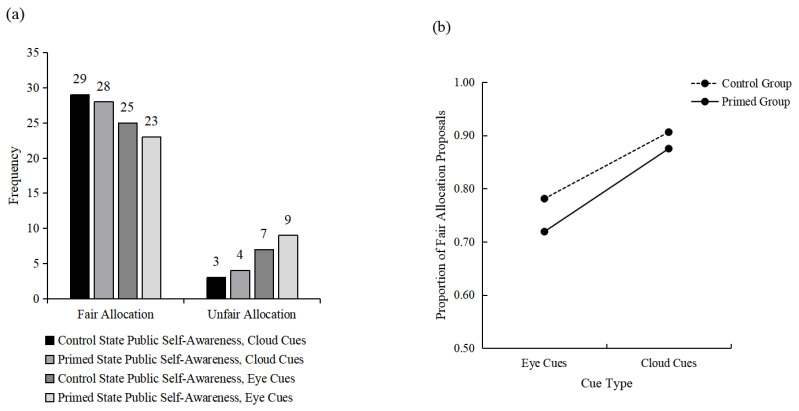
Study 2 interaction between cue type and proposers’ state public self-awareness (strict 5:5 allocation offers): (**a**) Interaction plot, (**b**) interaction line graph. Figure generated from Table 2 data (n=32 per cell, experimentally manipulated priming condition). The strict 5:5 counts: Cloud/Control = 29, Cloud/Prime = 28, Eye/Control = 25, Eye/Prime = 23.

For visualization, the Study 2 strict 5:5 allocation interaction pattern is shown in Figure 6.

#### 3.2.4. Effects on Responders’ Rejection of Non-5:5 Offers

Responders’ rejection of non-5:5 offers was coded from actual rejection decisions rather than from the allocation value alone. The rejection proportion was M=0.03, SD=0.18 in the cloud-cue condition and M=0.08, SD=0.27 in the eye-cue condition. The main effect of cue type was not significant, F(1, 124)=1.35, p=0.248, $\eta_{p}^{2}=0.011$. The main effect of state public self-awareness priming was not significant, F(1, 124)=0.15, p=0.699, $\eta_{p}^{2}=0.001$. The cue type × state public self-awareness interaction was not significant, F(1, 124)=1.35, p=0.248, $\eta_{p}^{2}=0.011$.

**Figure 7:**
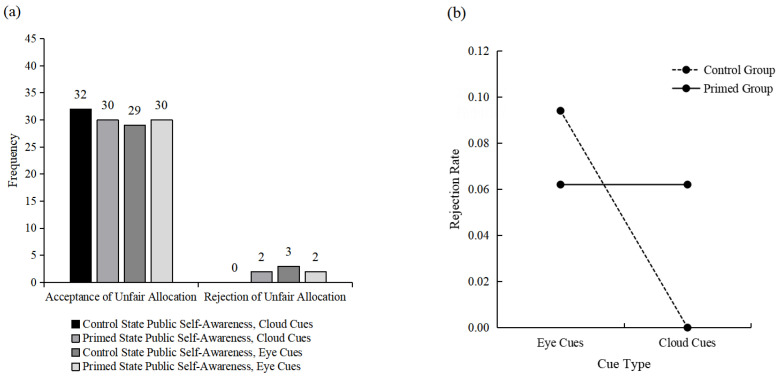
Study 2 interaction between cue type and responders’ state public self-awareness (rejection of non-5:5 offers): (**a**) Interaction plot, (**b**) interaction line graph. Figure generated from Table 2 data (n=32 per cell). The rejection counts: Cloud/Control = 0, Cloud/Prime = 2, Eye/Control = 3, Eye/Prime = 2 (total = 7; prior submission erroneously showed total = 5). Legend now includes all four experimental conditions.

For visualization, the Study 2 responder rejection interaction pattern is shown in Figure 7.

#### 3.2.5. Multiplicity Assessment and Logistic Robustness Check

Across the two studies, 30 focal inferential tests were conducted in total: Study 1 contributed 21 tests covering three dependent variables alongside seven predictive effects, including cue type, dispositional public self-awareness, decision round, and four pairwise and three-way associations among these variables, and Study 2 contributed nine tests covering three dependent variables alongside three predictive effects, including cue type, state public self-awareness priming, and the association between these two variables. Of these 30 tests, the cue-type effect on proposers’ strict 5:5 allocation offers in Study 2 was the only raw *p*-value below 0.05 (p=0.040). Under Holm stepdown family-wise error rate standard, the rank-1 threshold for 30 tests is $\alpha^{*}/k=0.05/30=0.0017$; p=0.040 does not meet this threshold. Benjamini–Hochberg false-discovery-rate standard yields the same conclusion: the FDR threshold for rank-1 is (1/30)×0.05=0.0017, which p=0.040 also exceeds. The single raw significant effect therefore does not stand up against repeated comparison standards.

As a robustness check, the binary strict 5:5 outcome was also analyzed using logistic models. First, a cue-only logistic regression (cloud cues = 0, eye cues = 1) showed that cloud-cue proposers made strict 5:5 offers on 57 of 64 trials (89.1%), whereas eye-cue proposers did so on 48 of 64 trials (75.0%). The model yielded $\hat{\beta }=-1.00$ (SE=0.49), z=−2.02, p=0.043, OR=0.37, 95% CI [0.14, 0.97]. This raw *p*-value closely mirrors the ANOVA result and likewise fails the Holm reference threshold ($\alpha^{*}=0.0017$). Second, a factorial logistic regression including cue type, state public self-awareness priming, and their interaction did not preserve a significant cue-type coefficient prior to evaluation ($\hat{\beta }=-1.00$, SE=0.74, z=−1.34, p=0.180), and the cue type × state public self-awareness interaction was also not significant (p=0.990). A dyad-level random-intercept mixed-effects logistic model was considered but was not substantively estimable because each dyad contributed exactly one proposer observation to this endpoint; the dyad-level variance component was therefore not identifiable. Thus, when the binary outcome was examined with models appropriate for dichotomous data, the single raw ANOVA effect did not provide reliable evidence for a cue-type effect.

### 3.3. Discussion

The Study 2 findings support a predominantly null interpretation. Cue type did not significantly affect proposers’ mean allocation amount or responders’ actual rejection of non-5:5 offers. State public self-awareness and its interaction with cue type were not significant for any focal behavioral outcome. Therefore, the previously described marginal interaction involving state public self-awareness should not be retained. The tentative trend observed for exact equal allocation disappeared after multiple comparison adjustment, and further binary outcome validation analysis also failed to confirm this pattern.

Importantly, the direction of the raw trend on the strict 5:5 outcome was opposite to the prosocial-surveillance prediction. Proposers under eye cues were descriptively *less* likely to make exact equal splits than proposers under cloud cues (75.0% vs. 89.1%). Three tentative contextual explanations are possible, though they should be treated with caution because the underlying effect was not reliable: (1) In anonymous online interactions, eye images may be processed as decorative interface elements rather than credible observation signals, attenuating any sense of being watched. (2) Under conditions of weak perceived observability, eye cues could incidentally activate competitive self-presentation motives rather than cooperative ones. (3) The boundary conditions of eye-cue effects may differ substantially between visible offline and anonymous online contexts. These explanations remain speculative; the present data do not provide reliable evidence for any directional effect, and the pattern should be treated only as a tentative descriptive anomaly rather than as evidence for a reversed directional effect.

## 4. General Discussion

The analyses yield a predominantly null pattern and require correspondingly cautious interpretation. Multiple behavioral indicators were measured across the two studies, and only one tentative raw trend appeared; this trend contradicted the common prosocial prediction and failed to hold after statistical adjustment for multiple comparisons, with additional supplementary analyses also failing to confirm the pattern. Study 1 showed no meaningful cue-related shift for any fairness indicator. Study 2 only contained this single unadjusted weak tendency related to equal division choices, which cannot be treated as reliable evidence.

Together with Study 1, Study 2 indicates that eye-cue effects in the online Ultimatum Game are not robust (Dear et al., 2019; Northover et al., 2017a, 2017b; Rotella et al., 2021; Tane & Takezawa, 2011). The absence of any effect standing against multiple comparison standards, combined with the unexpected direction of the raw trend, suggests that the present findings are more consistent with a null-dominant pattern than with the reliable watching-eyes effect on fair decision-making.

Therefore, the present findings should be interpreted as a transparent null-dominant contribution: two online Ultimatum Game studies that mostly failed to find supportive evidence for the watching-eyes effect on fair decision-making.

### 4.1. The Watching-Eyes Effect

The most important contribution of the findings is that they provide a transparent null-dominant test of the watching-eyes effect in online Ultimatum Game fairness decisions. No consistent cue-related tendency remained after comprehensive statistical adjustment, and the sole unadjusted weak trend ran counter to mainstream prosocial predictions. These findings suggest that eye cues cannot reliably alter people’s fairness choices under anonymous online conditions. This null-dominant pattern is broadly consistent with meta-analytic, field, and replication evidence showing that artificial surveillance cues do not uniformly increase prosocial behavior (Bateson et al., 2013; Dear et al., 2019; Fehr & Schneider, 2010; Northover et al., 2017a, 2017b; Oda, 2019; Rotella et al., 2021; Sparks & Barclay, 2013, 2015). The present studies contribute to this ongoing academic debate by separately testing three distinct fairness indicators, yet still found no stable influence of eye cues in online bargaining tasks.

### 4.2. Role of Public Self-Awareness

A second contribution concerns public self-awareness (Fenigstein et al., 1975). Neither dispositional public self-awareness in Study 1 nor experimentally primed state public self-awareness in Study 2 reliably moderated cue-type effects (Brockner et al., 1982; Keller & Pfattheicher, 2011; Pfattheicher & Keller, 2015; Van Bommel et al., 2012). In Study 1, the interaction between cue type and dispositional public self-awareness, the interaction between cue type and round, and the three-way interaction across all three variables failed to reach statistical significance for every measured behavioral outcome. In Study 2, the interaction between cue type and state public self-awareness also showed no meaningful effect for all focal indicators. These consistent null interactions suggest that public self-awareness was not a robust boundary condition for eye-cue effects in the present online Ultimatum Game context.

### 4.3. Theoretical and Practical Implications

Theoretically, the present findings suggest that the watching-eyes effect should be understood as conditional rather than universal. Eye cues may not automatically activate fairness norms in anonymous online bargaining contexts (Fehr & Schneider, 2010; Ikuse et al., 2018; Lamba & Mace, 2010). Their effects may depend on the credibility of observation, the decision role, and the outcome measure. Practically, this means that eye-like visual cues should not be used as stand-alone interventions for improving fairness in resource-allocation settings. Such cues may have weak or inconsistent effects unless the context makes social evaluation credible and salient, as in some offline or field settings where observation cues are more directly tied to reputational consequences (Bateson et al., 2013; Ernest-Jones et al., 2011; Francey & Bergmüller, 2012; Nettle et al., 2012).

Proposers and responders face different decision problems: proposers decide how to divide resources, whereas responders decide whether to accept or punish a proposed division (Oosterbeek et al., 2004; Sanfey et al., 2003). Across both studies, cue type did not affect responders’ rejection behavior once choices were coded according to actual decisions. No consistent cue-related tendency emerged for punitive responses to unequal offers in either experiment, which represents one of the most steady results observed in this research.

Although the studies did not yield a consistent significant cue-type effect, they produced two consistent null patterns that are theoretically meaningful. First, cue type did not significantly affect proposers’ mean allocation amount in either study. Second, cue type did not significantly affect responders’ rejection of non-5:5 offers in either study. These convergent null findings suggest that eye cues did not broadly alter the magnitude of resource allocation or actual punishment of unequal offers. Instead, any cue-related effect appears to be narrow and dependent on how fairness is operationalized.

### 4.4. Limitations and Future Directions

Several limitations should be noted. First, both studies were conducted online, where perceived observability may be weaker than in face-to-face contexts (Lamba & Mace, 2010). Second, strict 5:5 allocation offers capture exact equality but do not fully represent differences between near-equal and highly unequal offers (Sanfey et al., 2003). Third, although supplementary verification analyses were carried out for equal allocation choices in this research, the analytical framework could not form stable estimates based on the pairing structure of participants. Future work should complete detailed statistical plans in advance before data collection to avoid flexible analytical choices. Future studies should also directly compare continuous allocation amount, categorical equality choices, and responder rejection within the same design to clarify which aspects of fairness are most sensitive to visual social-presence cues. Given the predominantly null results in the present work, future studies using larger samples, online platforms with stronger perceived observation, or additional manipulations of social evaluation salience (Burnham & Hare, 2007; Mifune et al., 2010) would help clarify whether any watching-eyes effect can be reliably induced in online economic games. In summary, the findings do not support a reliable cue-type effect. Study 1 showed no meaningful cue-related tendency for any focal outcome. Study 2 only contained a single unadjusted weak tendency related to equal division choices that ran counter to the prosocial prediction and disappeared after multiple comparison adjustment. Across both studies, cue type did not shift proposers’ average allocation or responders’ rejection behavior, and public self-awareness could not act as a stable moderator for eye cues.

## 5. Conclusions

This research examined whether eye cues influence fair decision-making in the online Ultimatum Game and whether dispositional or state public self-awareness qualifies these effects. Study 1 found no meaningful influence of visual eye cues on proposers’ average resource offers, exact equal splits, or responders’ rejection of unequal proposals. Study 2 only displayed a weak raw trend related to equal allocation choices, which contradicted the mainstream prosocial prediction. This tentative pattern disappeared after accounting for multiple statistical comparisons, and additional binary outcome robustness analysis also failed to validate this tentative trend. Eye cues showed no consistent impact on average offers or responders’ punitive choices in Study 2. Neither trait nor situational public self-awareness acted as a reliable boundary factor to change how eye cues shaped participants’ fairness decisions. Taken together, the findings yield a predominantly null pattern. The contribution of the present work is a transparent null-dominant test showing that online eye cues may be too fragile to support strong claims about fairness interventions in anonymous Ultimatum Game contexts, which informs ongoing debate about the replicability and boundary conditions of the watching-eyes effect.

## Figures and Tables

**Table 1:** Descriptive statistics for dependent variables by cue type, trait public self-awareness, and round in Study 1.

Round	Cue Type	Trait Public Self-Awareness	Proposer				Responder		
			*n*	Allocation M(SD)	Strict 5:5 Trials/Total *n*	Strict 5:5 Proportion M(SD)	*n*	Rejection Trials/Total *n*	Rejection Rate M(SD)
R1	Cloud	Low	23	5.43 (0.90)	18/23	0.78 (0.42)	23	3/23	0.13 (0.34)
R1	Cloud	High	42	5.57 (1.29)	28/42	0.67 (0.48)	42	3/42	0.07 (0.26)
R1	Eye	Low	27	5.48 (1.40)	19/27	0.70 (0.47)	23	2/23	0.09 (0.29)
R1	Eye	High	38	5.84 (1.39)	23/38	0.61 (0.50)	42	5/42	0.12 (0.33)
R2	Cloud	Low	23	5.17 (0.78)	18/23	0.78 (0.42)	23	1/23	0.04 (0.21)
R2	Cloud	High	42	5.71 (1.50)	31/42	0.74 (0.45)	42	3/42	0.07 (0.26)
R2	Eye	Low	27	5.19 (1.24)	21/27	0.78 (0.42)	23	1/23	0.04 (0.21)
R2	Eye	High	38	5.61 (1.70)	26/38	0.68 (0.47)	42	3/42	0.07 (0.26)

**Table 2:** Descriptive statistics for dependent variables by cue type and state public self-awareness in Study 2.

Cue Type	State Public Self-Awareness	Proposer				Responder		
		*n*	Allocation M(SD)	Strict 5:5 Trials/Total *n*	Strict 5:5 Proportion M(SD)	*n*	Rejection Trials/Total *n*	Rejection Rate M(SD)
Cloud	Control	32	5.12 (0.42)	29/32	0.91 (0.30)	32	0/32	0.00 (0.00)
Cloud	Prime	32	5.33 (0.95)	28/32	0.88 (0.34)	32	2/32	0.06 (0.25)
Eye	Control	32	5.19 (1.06)	25/32	0.78 (0.42)	32	3/32	0.09 (0.30)
Eye	Prime	32	5.19 (0.93)	23/32	0.72 (0.46)	32	2/32	0.06 (0.25)

## Data Availability

To ensure full reproducibility, the de-identified raw data, cleaned analysis datasets, Study 2 session-to-condition mapping file, R analysis scripts for Study 1 and Study 2, and Supplementary Table S1 with statistical outputs are available in the OSF repository: rovide the date you accessed the URL in the following format: https://osf.io/n5jpk/files/osfstorage (accessed on 9 July 2026).

## Supplementary Materials

The following supporting information can be downloaded at: https://www.mdpi.com/article/10.3390/bs16071177/s1, Table S1: Descriptive and inferential statistics for Study 1 and Study 2; Study 1 Data: De-identified raw data, cleaned analysis data, corrected descriptive/inferential statistics, and mixed-design ANOVA analysis code; Study 2 Data: De-identified raw data, cleaned analysis data, corrected descriptive/inferential statistics, session-to-condition mapping, state public self-awareness descriptives, manipulation check data, and two-way ANOVA analysis code.
